# Supplementary material for: AIDmut-Seq: a Three-Step Method for Detecting Protein-DNA Binding Specificity
Source: Microbiol Spectr. 2022 Dec 19;11(1):e03783-22. doi: 10.1128/spectrum.03783-22 (PMC9927353; doi:10.1128/spectrum.03783-22)
Supplement: Supplemental file 1 — Supplemental material. Download spectrum.03783-22-s0001.pdf, PDF file, 0.7 MB [file spectrum.03783-22-s0001.pdf]

Supplementary Text

AIDMut-Seq: A three-step method for detecting protein-DNA binding specificity

Feixuan Li [a], Xiao-Yu Liu [c], Lei Ni [b,#], Fan Jin [b,#]

a. Hefei National Research Center for Physical Sciences at the Microscale; Department of Polymer Science and Engineering, University of Science and Technology of China, Hefei 230026, China
b. CAS Key Laboratory of Quantitative Engineering Biology, Shenzhen Institute of Synthetic Biology, Shenzhen Institutes of Advanced Technology, Chinese Academy of Sciences, Shenzhen 518055, China
c. School of Medicine, Southern University of Science and Technology, Shenzhen 518055, China

#, Address correspondence to Lei Ni, lei.ni@siat.ac.cn, or Fan Jin, fan.jin@siat.ac.cn.

Supplementary methods

Construction of gene deletion mutants in *P. aeruginosa*. PCR was used to generate 1000 bp DNA fragments upstream (Up) or downstream (Dn) from the *lasI*, *rhlI*, *lasR*, and *ung* genes. The Up and Dn DNA fragments for *lasI*, *rhlI*, *lasR*, and *ung* were ligated together using overlap extension PCR, and then inserted into the pex18gm vector via Gibson assembly. The recombinant plasmids were introduced into *P. aeruginosa* through electroporation, and the deletion mutants were obtained by double selection on LB agar supplemented with gentamycin (30 μg/mL) and NaCl-free LB agar containing 15% sucrose at 37 °C[1].

Whole genome sequencing and SNP acquisition. The genomic DNA (500 ng) were diluted into 130 μL elution buffer and disrupted via sonication into 500 bp fragments. DNA end-repair, A-base addition, adaptor ligation, purification, and amplification were performed with the Hieff NGS® MaxUp II DNA Library Prep Kit for Illumina. Sequencing was conducted on an Illumina Hiseq™ instrument. The original sequencing image data were transformed into raw reads with CASAVA and saved as FASTQ file. The FASTQ data files were filtered using Trimmomatic software to eliminate low-quality reads. Then, the filtered FASTQ data were compared with reference genome sequence (www.pseudomonas.com) using BWA software, and the comparison results were sorted and format-converted using SAMtools. The GATK toolkit was used to search and annotate SNPs in the genome.

Protein purification. Codon optimized (according to *Escherichia Coli*) *lasR* gene, and the original sequences of *fleQ* and *erdR* genes was cloned into the pET28a vector

and introduced into the BL21-RIL strain, generating the *TF*-BL21 strain. For LasR purification, single colony of *lasR*-BL21 was inoculated into LB broth supplemented with 50 μg/mL Kanamycin, 37 μg/mL chloramphenicol and 1 μM C_{12} at 37 °C until OD~0.5. Bacterial culture was cooled down on ice, then 0.1 mM IPTG were added and the culture were incubated for another 20 hours at 16 °C. Bacterial cells were harvested via centrifugation and the pellets were resuspended in the lysis buffer (20mM Tris 8.0， 300mM NaCl， 1mM DTT， 20mM imidazole). Ultrasonication was performed under 20% power and pulse on 1s off 2s for 10 minutes. Protein supernatant was obtained by centrifuging 12,000 rpm, 30 min, at 4˚C and injected into a Ni-NTA column. The Ni-NTA column was washed with 40 mM imidazole and LasR was eluted with 400 mM imidazole elute (imidazole was prepared with the same lysis buffer mentioned above). Then the elution fractions were dialyzed against buffer (20mM Tris-HCl pH 8.0, 150 mM NaCl, 1mM DTT), and applied onto 5 mL HiTrap Q Fastflow column (GE Healthcare). LasR were eluted by a linear gradient from 100 mM NaCl to 1 M NaCl in 1.8mL. Fractions were then concentrated in 10 kDa molecular mass cut-off concentrators (Amicon) to about 1mL and further purified by Superdex 75 Increase 10/300 gel filtration column equilibrated in the buffer containing 20 mM Tris-HCl pH 8.0, 150 mM NaCl, 1 mM DTT. The peak fractions were collected for further procedures. Experimental procedures for ErdR and FleQ purification were without the addition of C_{12} during TF-BL21 culture. ErdR was purified without dialysis, simply by washing through a desalination column after 400

mM imidazole elution. FleQ purification was conducted following the same protocol for LasR purification.

Quantification of the intracellular expression level of AID-TF. We use the fluorescent protein reporter based method as described previously to quantify the expression of the arabinose-inducible promoter on pJN105 [2]. Briefly, an *sfGFP*-pJN105 plasmid, in which SfGFP was expressed by the same arabinose-inducible promoter as AID-TF, was constructed and introduced to the PAO1Δ*ung* strain. Bacteria were cultured in the same media and following the same procedure as AIDmut-Seq experiment. Then, bacterial cells of 12-hour induction by 0.4%, 0.1%, 0.05% arabinose were observed under microscope. Fluorescent images of SfGFP were captured using Zyla 4.2 sCMOS camera (Andor) on a microscope (IX-71, Olympus) equipped with a 100× oil objective. The fluorescence of SfGFP was excited at 488 nm using a solid-state light source (Lumencor Spectra X), and collected with emission filters of 520/28 nm. Image processing was conducted under the MATLAB environment with a self-written code. Single-cell intensities of SfGFP were determined according to cell masks using a built-in function (regionprops). In addition, the protein concentration of SfGFP were determined according to the concentration-intensity relationship of purified SfGFP protein. Computer codes are available on request.

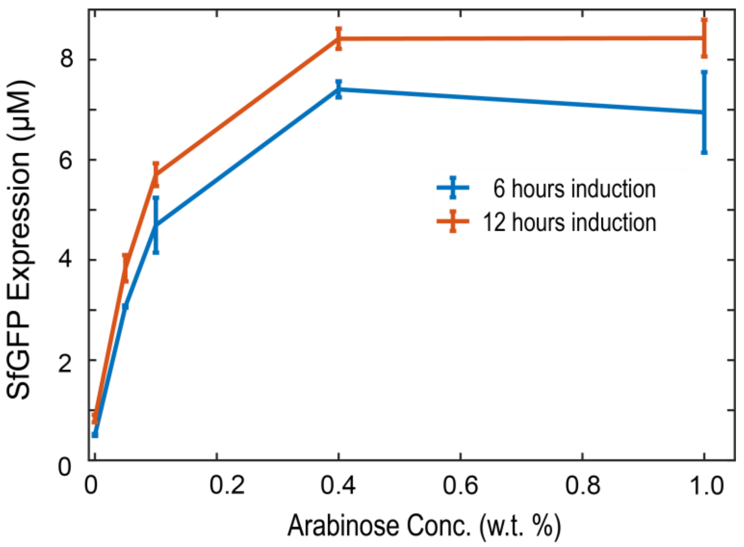

Figure S1. Expression level of SfGFP at different arabinose concentration. *sfGFP* was expressed by the arabinose-inducible promoter pBAD in the pJN105 plasmid.

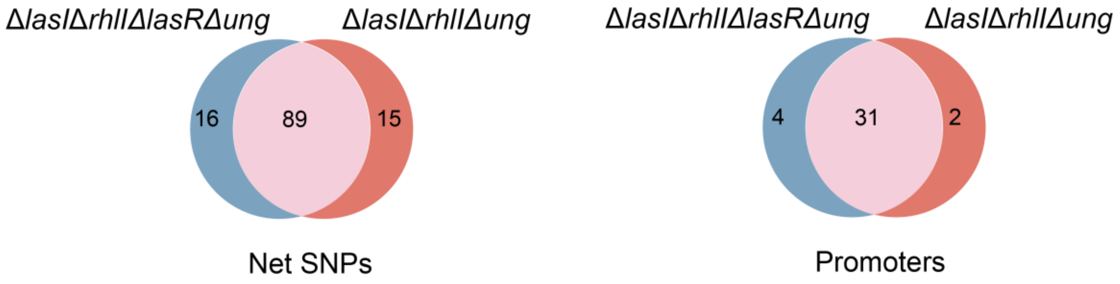

Figure S2. Comparation of AIDmut-Seq detected net SNPs for LasR in the Δ*lasI*Δ*rhlI*Δ*lasR*Δ*ung* quadruple mutant or the Δ*lasI*Δ*rhlI*Δ*ung* triple mutant strains (left), promoters corresponding to those SNPs are shown on the right.

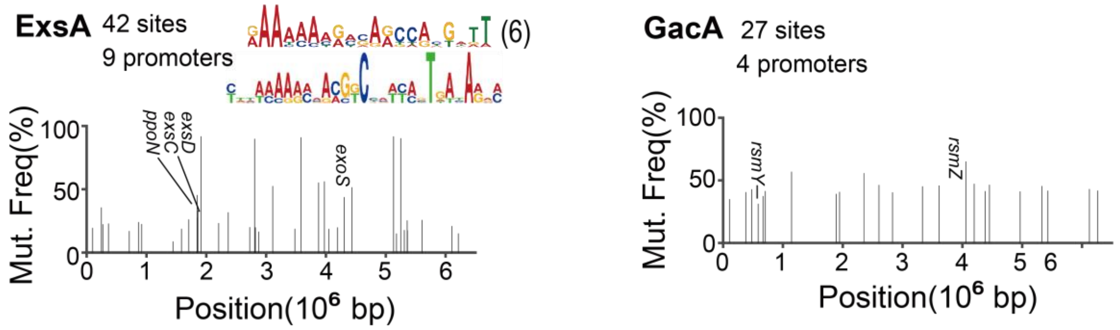

Figure S3. AIDmut-Seq SNP spectrum of ExsA and GacA.

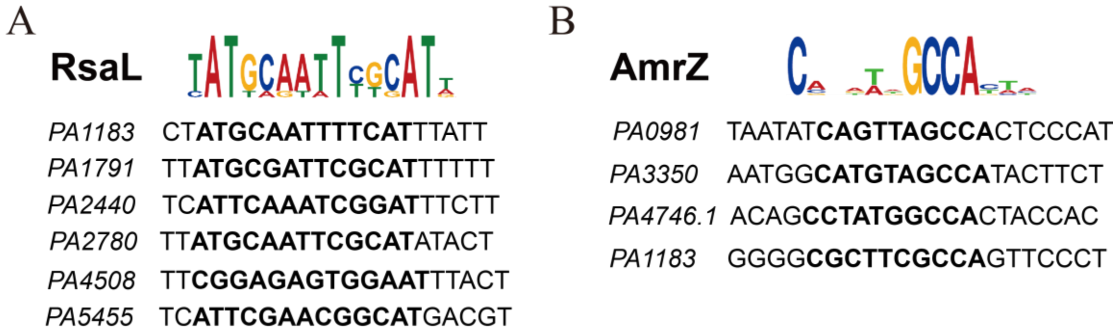

Figure S4. Mapping of RsaL (A) or AmrZ binding consensus at the newly found promoter sequences. The putative TF-binding sequences in each promoter were displayed in bold.

DNA sequences of genes or vector used in the study.

pJN105 vector (pBAD promoter sequence was shown in bold, black arrow indicate the direction of transcription, the inverted triangle indicates the position of insertion for the expression of *AID-TF* fusion genes)

accttcgggagcgcctgaagcccgttctggacgccctggggccgttgaatcgggatatgcaggccaaggccgccgcgatcatcaaggccg
tgggcgaaaagctgctgacggaacagcgggaagtccagcgccagaaacaggcccagcgccagcaggaacgcgggcgcgcacatttcc
ccgaaaagtgccacctggcggcgttgtgacaatttaccgaacaactccgcggccgggaagccgatctcggcttgaacgaattgttaggtggc
ggtacttgggtcgatatcaaagtgcatcacttcttcccgtatgcccaactttgtatagagagccactgcgggatcgtcaccgtaatctgcttgcac
gtagatcacataagcaccaagcgcgttggcctcatgcttgaggagattgatgagcgcggtggcaatgccctgcctccggtgctcgccggaga
ctgcgagatcatagatatagatctcactacgcggctgctcaaacctgggcagaacgtaagccgcgagagcgccaacaaccgcttcttggtcg
aaggcagcaagcgcgatgaatgtcttactacggagcaagttcccgaggtaatcggagtccggctgatgttgggagtaggtggctacgtctcc
gaactcacgaccgaaaagatcaagagcagcccgcatggatttgacttggtcagggccgagcctacatgtgcgaatgatgcccatacttgagc
cacctaactttgttttagggcgactgccctgctgcgtaacatcgttgctgctgcgtaacatcgttgctgctccataacatcaaacatcgacccacg
gcgtaacgcgcttgctgcttggatgcccgaggcatagactgtacaaaaaaacagtcataacaagccatgaaaaccgccactgcgccgttacc
accgctgcgttcggtcaaggttctggaccagttgcgtgagcgcatacgctacttgcattacagtttacgaaccgaacaggcttatgtcaactgg
gttcgtgccttcatccgtttccacggtgtgcgtccatgggcaaatattatacgcaaggcgacaaggtgctgatgccgctggcgattcaggttcat
catgccgtttgtgatggcttccatgtcggcagaatgcttaatgaattacaacagtttttatgcatgcgcccaatacgcaaaccgcctctccccgcg
cgttggccgattcattaatgcagctggcacgacaggtttcccgactggaaagcgggcagtgagcgcaacgcaattaatgtgagttagctcact
cattaggcaccccaggctttacactttatgcttccggctcgtatgttgtgtggaattgtgagcggataacaatttcacacaggaaacagctatgac
catgattacgccaagcgcgcaattaaccctcactaaagggaacaaaagctgggtaccgggccccccctcgaggtcgacggtatcgatgcat
aatgtgcctgtcaaatggacgaagcagggattctgcaaaccctatgctactccgtcaagccgtcaattgtctgattcgttaccaattatgacaact
tgacggctacatcattcactttttcttcacaaccggcacggaactcgctcgggctggccccggtgcattttttaaatacccgcgagaaatagagtt
gatcgtcaaaaccaacattgcgaccgacggtggcgataggcatccgggtggtgctcaaaagcagcttcgcctggctgatacgttggtcctcg
cgccagcttaagacgctaatccctaactgctggcggaaaagatgtgacagacgcgacggcgacaagcaaacatgctgtgcgacgctggcg
atatcaaaattgctgtctgccaggtgatcgctgatgtactgacaagcctcgcgtacccgattatccatcggtggatggagcgactcgttaatcgc
ttccatgcgccgcagtaacaattgctcaagcagatttatcgccagcagctccgaatagcgcccttccccttgcccggcgttaatgatttgcccaa
acaggtcgctgaaatgcggctggtgcgcttcatccgggcgaaagaaccccgtattggcaaatattgacggccagttaagccattcatgccagt
aggcgcgcggacgaaagtaaacccactggtgataccattcgcgagcctccggatgacgaccgtagtgatgaatctctcctggcgggaacag
caaaatatcacccggtcggcaaacaaattctcgtccctgatttttcaccaccccctgaccgcgaatggtgagattgagaatataacctttcattcc
cagcggtcggtcgataaaaaaatcgagataaccgttggcctcaatcggcgttaaacccgccaccagatgggcattaaacgagtatcccggca
gcaggggatcattttgcgcttcagccatacttttcatactcccgccattcagagaagaaaccaattgtccatattgcatcagacattgccgtcact
gcgtcttttactggctcttctcgctaaccaaaccggtaaccccgcttattaaaagcattctgtaacaaagcgggacc**aaagccatgacaaaaa**
cgcgtaacaaaagtgtctataatcacggcagaaaagtccacattgattatttgcacggcgtcacactttgctatgccatagcatttttat
ccataagattagcggatcctacctgacgctttttatcgcaactctctactgtttctccatacccgtttttttgggctagcgaattcgagctcca
attcgccctatagtgagtcgtattacgcgcgctcactggccgtcgttttacaacgtcgtgactgggaaaaccctggcgttacccaacttaatcgc
cttgcagcacatccccctttcgccagctggcgtaatagcgaagaggcccgcaccgatcgcccttcccaacagttgcgcagcctgaatggcga
atggaaattgtaagcgttaatattttgttaaaattcgcgttaaatttttgttaaatcagctcattttttaaccaataggccgactgcgatgagtggcag
ggcggggcgtaatttttttaaggcagttattggtgcccttaaacgcctggtgctacgcctgaataagtgataataagcggatgaatggcagaaat
tcgaaagcaaattcgacccggtcgtcggttcagggcagggtcgttaaatagccgcttatgtctattgctggtttaccggtttattgactaccggaa
gcagtgtgaccgtgtgcttctcaaatgcctgaggccagtttgctcaggctctccccgtggaggtaataattgacgatatgatcatttattctgcctc
ccagagcctgataaaaacggtgaatccgttagcgaggtgccgccggcttccattcaggtcgaggtggcccggctccatgcaccgcgacgca
acgcggggaggcagacaaggtatagggcggcgaggcggctacagccgatagtctggaacagcgcacttacgggttgctgcgcaacccaa

gtgctaccggcgcggcagcgtgacccgtgtcggcggctccaacggctcgccatcgtccagaaaacacggctcatcgggcatcggcaggc
gctgctgcccgcgccgttcccattcctccgtttcggtcaaggctggcaggtctggttccatgcccggaatgccgggctggctgggcggctcct
cgccggggccggtcggtagttgctgctcgcccggatacagggtcgggatgcggcgcaggtcgccatgccccaacagcgattcgtcctggt
cgtcgtgatcaaccaccacggcggcactgaacaccgacaggcgcaactggtcgcggggctggccccacgccacgcggtcattgaccacg
taggccgacacggtgccggggccgttgagcttcacgacggagatccagcgctcggccaccaagtccttgactgcgtattggaccgtccgca
aagaacgtccgatgagcttggaaagtgtcttctggctgaccaccacggcgttctggtggcccatctgcgccacgaggtgatgcagcagcatt
gccgccgtgggtttcctcgcaataagcccggcccacgcctcatgcgctttgcgttccgtttgcacccagtgaccgggcttgttcttggcttgaat
gccgatttctctggactgcgtggccatgcttatctccatgcggtagggtgccgcacggttgcggcaccatgcgcaatcagctgcaacttttcgg
cagcgcgacaacaattatgcgttgcgtaaaagtggcagtcaattacagattttctttaacctacgcaatgagctattgcgggggggtgccgcaat
gagctgttgcgtacccccctttttaagttgttgatttttaagtctttcgcatttcgccctatatctagttctttggtgcccaaagaagggcacccctgc
ggggttcccccacgccttcggcgcggctccccctccggcaaaaagtggcccctccggggcttgttgatcgactgcgcggccttcggccttgc
ccaaggtggcgctgccccttggaacccccgcactcgccgccgtgaggctcgggggggcaggcgggcgggcttcgccttcgactgccccc
actcgcataggcttgggtcgttccaggcgcgtcaaggccaagccgctgcgcggtcgctgcgcgagccttgacccgccttccacttggtgtcc
aaccggcaagcgaagcgcgcaggccgcaggccggaggcttttccccagagaaaattaaaaaaattgatggggcaaggccgcaggccgc
gcagttggagccggtgggtatgtggtcgaaggctgggtagccggtgggcaatccctgtggtcaagctcgtgggcaggcgcagcctgtccat
cagcttgtccagcagggttgtccacgggccgagcgaagcgagccagccggtggccgctcgcggccatcgtccacatatccacgggctggc
aagggagcgcagcgaccgcgcagggcgaagcccggagagcaagcccgtagggcgccgcagccgccgtaggcggtcacgactttgcg
aagcaaagtctagtgagtatactcaagcattgagtggcccgccggaggcaccgccttgcgctgcccccgtcgagccggttggacaccaaaa
gggaggggcaggcatggcggcatacgcgatcatgcgatgcaagaagctggcgaaaatgggcaacgtggcggccagtctcaagcacgcc
taccgcgagcgcgagacgcccaacgctgacgccagcaggacgccagagaacgagcactgggcggccagcagcaccgatgaagcgatg
ggccgactgcgcgagttgctgccagagaagcggcgcaaggacgctgtgttggcggtcgagtacgtcatgacggccagcccggaatggtg
gaagtcggccagccaagaacagcaggcggcgttcttcgagaaggcgcacaagtggctggcggacaagtacggggcggatcgcatcgtg
acggccagcatccaccgtgacgaaaccagcccgcacatgaccgcgttcgtggtgccgctgacgcaggacggcaggctgtcggccaagga
gttcatcggcaacaaagcgcagatgacccgcgaccagaccacgtttgcggccgctgtggccgatctagggctgcaacggggcatcgaggg
cagcaaggcacgtcacacgcgcattcaggcgttctacgaggccctggagcggccaccagtgggccacgtcaccatcagcccgcaagcgg
tcgagccacgcgcctatgcaccgcagggattggccgaaaagctgggaatctcaaagcgcgttgagacgccggaagccgtggccgaccgg
ctgacaaaagcggttcggcaggggtatgagcctgccctacaggccgccgcaggagcgcgtgagatgcgcaagaaggccgatcaagccc
aagagacggcccgag

***AID732-lasR* translational fusion** (*AID732* gene sequence was shown in blue, *lasR* gene sequence was shown in green, the Gly8 linker sequence between AID732 and LasR was shown in bold)

ATGGACAGCCTGCTGATGAACCGCCGCGAGTTCCTGTACCAGTTCAAGAACGTGCGCT
GGGCCAAGGGCCGCCGCGAGACCTACCTGTGCTACGTGGTGAAGCGCCGCGACAGCG
CCACCAGCTTCAGCCTGGACTTCGGCTACCTGCGCAACAAGAACGGCTGCCACGTGG
AGCTGCTGTTCCTGCGCTACATCACCGACTGGGACCTGGACCCGGGCCGCTGCTACCG
CGTGACCTGGTTCATCAGCTGGAGCCCGTGCTACGACTGCGCCCGCCACGTGGCCGAC
TTCCTGCGCGGCAACCCGAACATCAGCCTGCGCATCTTCACCGCCCGCCTGTACTTCTG
CGAGGACCGCAAGGCCGAGCCGGAGGGCCTGCGCCGCCTGCACCGCGCCGGCGTGC
AGATCGCCATCATGACCTTCAAGGACTACTTCTACTGCTGGAACACCTTCGTGGAGAA
CCACGGCCGCACCTTCGAAGCCTGGGAGGGCCTGCACGAGAACAGCGTGCGCCTGAG
CCGCCAGCTGCGCCGCATCCTG**GGTGGTGGCGGAGGCGGCGGTGGA**ATGGCCTTG
GTTGACGGTTTTCTTGAGCTGGAACGCTCAAGTGGAAAATTGGAGTGGAGCGCCATCC

TGCAGAAGATGGCGAGCGACCTTGGATTCTCGAAGATCCTGTTCGGCCTGTTGCCTAA
GGACAGCCAGGACTACGAGAACGCCTTCATCGTCGGCAACTACCCGGCCGCCTGGCG
CGAGCATTACGACCGGGCTGGCTACGCGCGGGTCGACCCGACGGTCAGTCACTGTACC
CAGAGCGTACTGCCGATTTTCTGGGAACCGTCCATCTACCAGACGCGAAAGCAGCACG
AGTTCTTCGAGGAAGCCTCGGCCGCCGGCCTGGTGTATGGGCTGACCATGCCGCTGCA
TGGTGCTCGCGGCGAACTCGGCGCGCTGAGCCTCAGCGTGGAAGCGGAAAACCGGGC
CGAGGCCAACCGTTTCATGGAGTCGGTCCTGCCGACCCTGTGGATGCTCAAGGACTAC
GCACTGCAGAGCGGTGCCGGACTGGCCTTCGAACATCCGGTCAGCAAACCGGTGGTT
CTGACCAGCCGGGAGAAGGAAGTGTTGCAGTGGTGCGCCATCGGCAAGACCAGTTGG
GAGATATCGGTTATCTGCAACTGCTCGGAAGCCAATGTGAACTTCCATATGGGAAATAT
TCGGCGGAAGTTCGGTGTGACCTCCCGCCGCGTAGCGGCCATTATGGCCGTTAATTTGG
GTCTTATTACTCTCTGA

AIDΔ

ATGGACAGCCTGCTGATGAACCGCCGCGAGTTCCTGTACCAGTTCAAGAACGTGCGCT
GGGCCAAGGGCCGCCGCGAGACCTACCTGTGCTACGTGGTGAAGCGCCGCGACAGCG
CCACCAGCTTCAGCCTGGACTTCGGCTACCTGCGCAACAAGAACGGCTGCCACGTGG
AGCTGCTGTTCCTGCGCTACATCAGCGACTGGGACCTGGACCCGGGCCGCTGCTACCG
CGTGACCTGGTTCATCAGCTGGAGCCCGTGCTACGACTGCGCCCGCCACGTGGCCGAC
TTCCTGCGCGGCAACCCGAACCTGAGCCTGCGCATCTTCACCGCCCGCCTGTACTTCT
GCGAGGACCGCAAGGCCGAGCCGGAGGGCCTGCGCCGCCTGCACCGCGCCGGCGTG
CAGATCGCCATCATGACCTTCAAGGACTACTTCTACTGCTGGAACACCTTCGTGGAGA
ACCACGGCCGCACCTTCAAGGCCTGGGAGGGCCTGCACGAGAACAGCGTGCGCCTGA
GCCGCCAGCTGCGCCGCATCCTGCTGCCGCTGTACGAGGTGGACGACCTGCGCGACGC
CTTCCGCACCTGA

AID731

ATGGACAGCCTGCTGATGAACCGCAGCGAGTTCCTGTACCAGTTCAAGAACGTGCGCT
GGGCCAAGGGCCGCCGCGAGACCTACCTGTGCTACGTGGTGAAGCGCTGCGACAGCG
CCACCAGCTTCAGCCGCGACTTCGGCTACCTGCGCAACAAGAACGGCTGCCACGTGG
AGCTGCTGTTCCTGCGCTACATCAGCGACTGGGACCTGGACCCGGGCCGCTGCTACCG
CGTGACCTGGTTCATCAGCTGGAGCCCGTGCAGCGACTGCGCCCGCCTGGTGGCCGAC
TTCCTGCGCGGCAACCCGAACCTGAGCCTGCGCATCTTCACCGCCCGCCTGTACTTCT
GCGAGGACCGCAAGGCCGAGCCGGAGGGCCTGCGCCGCCTGCACCGCGCCGGCGTG
CAGATCGCCATCATGACCTTCGAAGACTACTTCTACTGCTGGAACACCTTCGTGGAGA
ACCACGGCCGCACCTTCAAGGCCTGGGAGGGCCTGCACGAGAACAGCGTGCGCCTGA
GCCGCCAGCTGCGCCGCATCCTGCTGCCGCTGTACGAGGTGGACGACCTGCGCGACGC
CTTCCGCACCCTGGGCCTGTGA

AID733

ATGGACAGCCTGCTGATGAACCGCCGCGAGTTCCTGTACCAGTACAAGAACGTGCGCT
GGGCCAAGGGCCGCCGCGAGACCTACCTGTGCTACGTGGTGAAGCGCCGCGACAGCG
CCACCAGCTTCAGCCTGGACTTCGGCTACCTGCGCAACAAGAACGGCTGCCACGTGG
AGCTGCTGTTCCTGCGCTACATCAGCGACTGGGACCTGGACCCGGGCCGCTGCTACCG

CGTGACCTGGTTCATCAGCTGGAGCCCGTGCTACGACTGCGCCCGCCACGTGGCCGACTTCCTGCGCGGCAACCCGAACCTGAGCCTGCGCATCTTCACCGCCCGCCTGTACTATTGCGAGGACCGCAAGGCCGAGCCGGAGGGCCTGCGCCGCCTGCACCGCGCCGGCGTGCAGATCGCCATCATGACCTTCAAGGACTACTTCTACTGCTGGAACACCTTCGTGGAGAACCACGGCCGCACCTTCAAGGCCTGGGAGGGCCTGCACGAGAACAGCGTGCGCCTGAGCCGCCAGCTGCGCCGCATCCTGCTGCCGCTGTACGGCGTGGACGACCTGCGCGACGCCTTCCGCACCCTGGGCCTGTGA

Amino acid sequences used in this study

AID732-LasR (AID732 sequence was shown in blue, LasR sequence was shown in green, the Gly8 linker was shown in bold)

MDSLLMNRREFLYQFKNVRWAKGRRETYLCYVVKRRDSATSFSLDFGYLRNKNGCHVELLFLRYITDWDLDPGRCYRVTWFISWSPCYDCARHVADFLRGNPNISLRIFTARLYFCEDRKAEPEGLRRLHRAGVQIAIMTFKDYFYCWNTFVENHGRTFEAWEGLHENSVRLSRQLRRIL**GGGGGGGG**MALVDGFLELERSSGKLEWSAILQKMASDLGFSKILFGLLPKDSQDYENAFIVGNYPAAWREHYDRAGYARVDPTVSHCTQSVLPIFWEPSIYQTRKQHEFFEEASAAGLVYGLTMPLHGARGELGALSLSVEAENRAEANRFMESVLPTLWMLKDYALQSGAGLAFEHPVSKPVVLTSREKEVLQWCAIGKTSWEISVICNCSEANVNFHMGNIRRKFGVTSRRVAAIMAVNLGLITL

AIDΔ

MDSLLMNRREFLYQFKNVRWAKGRRETYLCYVVKRRDSATSFSLDFGYLRNKNGCHVELLFLRYISDWDLDPGRCYRVTWFISWSPCYDCARHVADFLRGNPNLSLRIFTARLYFCEDRKAEPEGLRRLHRAGVQIAIMTFKDYFYCWNTFVENHGRTFKAWEGLHENSVRLSRQLRRILLPLYEVDDLRDAFRT

AID731

MDSLLMNRSEFLYQFKNVRWAKGRRETYLCYVVKRCDSATSFSRDFGYLRNKNGCHVELLFLRYISDWDLDPGRCYRVTWFISWSPCSDCARLVADFLRGNPNLSLRIFTARLYFCEDRKAEPEGLRRLHRAGVQIAIMTFEDYFYCWNTFVENHGRTFKAWEGLHENSVRLSRQLRRILLPLYEVDDLRDAFRTLGL

AID733

MDSLLMNRREFLYQYKNVRWAKGRRETYLCYVVKRRDSATSFSLDFGYLRNKNGCHVELLFLRYISDWDLDPGRCYRVTWFISWSPCYDCARHVADFLRGNPNLSLRIFTARLYYCEDRKAEPEGLRRLHRAGVQIAIMTFKDYFYCWNTFVENHGRTFKAWEGLHENSVRLSRQLRRILLPLYGVDDLRDAFRTLGL

Table S1. Bacterial strains, plasmids and PCR primers

Strains

Description	Genotype or relevant phenotype	Source or reference
***E.coli* strains**		
TOP10	*endA hsdR17 supE44 thi-1 recA1 gyrA relA1 D* (*lacZYA-argF*) U169 *deoR* (ϕ80d*lacD* (*lacZ*) M15)	Invitrogen
BL21(RIL)	*E. coli B* F^- *ompT hsdS*(r_B^- m_B^-) dcm^+ Tet^r *gal λ(DE3) endA Hte [argU ileY leuW* Cam^r*]*	Agilent
***P. aeruginosa* strains**		
PAO1	wild type	Lab stock
PAO1-*Δung*	deletion of *ung*	This study
PAO1-*ΔrhlI-ΔlasI-Δung*	deletion of *rhlI*, *lasI*, and *ung*	This study
PAO1-*ΔrhlI-ΔlasI-Δung-ΔlasR*	deletion of *rhlI*, *lasI*, *lasR* and *ung*	This study
PAO1-*ΔfleQ*	deletion of *fleQ*	This study
PAO1-*ΔerdR*	deletion of *erdR*	This study

Plasmids

Plasmids	Description	Source or reference
pJN105	Shuttle plasmid with ParaBAD promoter for gene expression under *L*-arabinose inducing, Gen^r	Lab stock
AIDΔ-pJN105	PJN105 containing *AIDΔ* ORF	This study
AIDΔ-lasR-pJN105(nl)	PJN105 containing translational translational fusion ORF of *AIDΔ* and *lasR*, with no linker	This study
AIDΔ-lasR-pJN105(G2)	PJN105 containing translational fusion ORF of *AIDΔ* and *lasR*, with G2 linker	This study
AIDΔ-lasR-pJN105(G4)	PJN105 containing translational fusion ORF of *AIDΔ* and *lasR*, with G4 linker	This study
AIDΔ-lasR-pJN105(G8)	PJN105 containing translational fusion ORF of *AIDΔ* and *lasR*, with G8 linker	This study
AIDΔ-lasR-pJN105((G_4S)1)	PJN105 containing translational fusion ORF of *AIDΔ* and *lasR*, with (G_4S)1 linker	This study
AIDΔ-lasR-pJN105((G_4S)3)	PJN105 containing translational fusion ORF of *AIDΔ* and *lasR*, with (G_4S)3 linker	This study
AIDΔ-lasR-pJN105((G_4S)5)	PJN105 containing translational fusion ORF of *AIDΔ* and *lasR*, with (G_4S)5 linker	This study

AIDΔ-*lasR*-pJN105((EA$_3$K)1)	PJN105 containing translational fusion ORF of *AIDΔ* and *lasR*, with (EA$_3$K)1 linker	This study
AIDΔ-*lasR*-pJN105((EA$_3$K)3)	PJN105 containing translational fusion ORF of *AIDΔ* and *lasR*, with (EA$_3$K)3 linker	This study
AIDΔ-*lasR*-pJN105((EA$_3$K)5)	PJN105 containing translational fusion ORF of *AIDΔ* and *lasR*, with (EA$_3$K)5 linker	This study
lasR-*AIDΔ*-pJN105	PJN105 containing translational fusion ORF of *lasR* and *AIDΔ*, with no linker	This study
AID731-*lasR*-pJN105	PJN105 containing translational fusion ORF of *AID731* and *lasR*, with G8 linker	This study
AID732-*lasR*-PJN105	PJN105 containing translational fusion ORF of *AID732* and *lasR*, with G8 linker	This study
AID733-*lasR*-pJN105	PJN105 containing translational fusion ORF of *AID733* and *lasR*, with G8 linker	This study
AID732-pJN105	PJN105 containing *AID732* ORF	This study
AID732-*fleQ*-pJN105	PJN105 containing translational fusion ORF of *AID732* and *fleQ*, with G8 linker	This study
AID732-*exsA*-pJN105	PJN105 containing translational fusion ORF of *AID732* and *exsA*, with G8 linker	This study
AID732-*amrZ*-pJN105	PJN105 containing translational fusion ORF of *AID732* and *amrZ*, with G8 linker	This study
AID732-*erdR*-pJN105	PJN105 containing translational fusion ORF of *AID732* and *erdR*, with G8 linker	This study
AID732-*gntR*-pJN105	PJN105 containing translational fusion ORF of *AID732* and *gntR*, with G8 linker	This study
AID732-*rsaL*-pJN105	PJN105 containing translational fusion ORF of *AID732* and *rsaL*, with G8 linker	This study
AID732-*gacA*-pJN105	PJN105 containing translational fusion ORF of *AID732* and *gacA*, with G8 linker	This study
AID732-*hmgR*-pJN105	PJN105 containing translational fusion ORF of *AID732* and *hmgR*, with G8 linker	This study
pET28a	For 6xHis-tag protein expression in *E. coli*,Kan^r	Invitrogen
pET28a-*lasR*	pET28a containing *lasR* ORF	This study
pET28a-*erdR*	pET28a containing *erdR* ORF	This study
pET28a-*fleQ*	pET28a containing *fleQ* ORF	This study

Primers-EMSA primers

ErdR

**Primers**	**Sequence (5'-3')**	
0045_EMSA-F	GTCGACGGCAGAGACGGAC	This study
0045_EMSA-R	CGCTGTGACTCATGGGGTTCTC	This study
0508_EMSA-F	GAGCAGTTTTCGCTCTAGCGGC	This study
0508_EMSA-R	CAGCCATGTGGGGGAATCCTC	This study

1182/83_EMSA-F	GCGGCGTCCTTGTTGTTGG	This study
1182/83_EMSA-R	GGTTGTTTGGTCATGGGCAGAG	This study
2623/24_EMSA-F	CATGCTGAACTCCGTGTTGTGG	This study
2623/24_EMSA-R	CGTTGTACGGCGGGTTCATG	This study
2679/80_EMSA-F	GACTCTCCCGAAATGATCCCGG	This study
2679/80_EMSA-R	GTGATGCGACTGGGCATGG	This study
3235_EMSA-F	CGCTGGTAGATGCTGTCGTTC	This study
3235_EMSA-R	CAGTAGCACGGCTGGTTCGATC	This study
3266_EMSA-F	CATCGAATATGCTCCGAGGGGT	This study
3266_EMSA-R	GACGATTCGACATGGTGTAACTCCTG	This study
3437_EMSA-F	GAAGCGCACTGACGGCTTG	This study
3437_EMSA-R	CATGGGACTCTCACAGCAGGC	This study
3568_EMSA-F	GTGCTGCTGCAGGTACGTCA	This study
3568_EMSA-R	GCTGGACTTCTCCAGCATCCAG	This study
3766_EMSA-F	CACTCATGACAACAACTCCCTGC	This study
3766_EMSA-R	GAAGCGGCGAAGAAGCCTC	This study
3986/87_EMSA-F	GCAAGCTTAGCGTTCCCGTTC	This study
3986/87_EMSA-R	TAGCTCCCTGACGAGGAAGGAC	This study
0887_EMSA-F	GCTGGGCATGAGCTGAGGTC	This study
0887_EMSA-R	GGCTTTAACCTCGGTACGTGAG	This study
4606_EMSA-F	CGAAAATTCTAGCCGCGAACCG	This study
4606_EMSA-R	CGAAGTGTTTTCGCCTGGCC	This study
1755_EMSA-F	GACTTCCTTGCACTTGCGGC	This study
1755_EMSA-R	GAAGCCACCCACAAGGAGTGC	This study

FleQ

**Primers**	**Sequence (5'-3')**	
0359_EMSA-F	GCCTGATCGGCTTCTACCGG	This study
0359_EMSA-R	CGAAACAGGTTCATGGAGGCG	This study
0362_EMSA-F	CTAACAACTCCAACCGCGACG	This study
0362_EMSA-R	CGTTTCAAGCGCTGATCCGC	This study
2392/93_EMSA-F	CATGAATCCGCGTCGAGAAACC	This study
2392/93_EMSA-R	GAGCGGGAAAATTAGCGCCG	This study
2619_EMSA-F	CACCTGATAGCCGTGTGGCG	This study
2619_EMSA-R	GCAGTAGAGCCCTCGGTATCTATG	This study
2653/54_EMSA-F	GCACCACTGCGTTCATCGTTG	This study
2653/54_EMSA-R	CGAAGGAACATGGGAGGGGTC	This study
2955_EMSA-F	GCTTCATGCAGGCGGACAG	This study
2955_EMSA-R	CTTTGATGGCGCGGAAGCTG	This study
4583_EMSA-F	GCCTGGACCAGGTATTGAACGG	This study
4583_EMSA-R	CCTGGGTCCAGAGCTTGATCG	This study
4958_EMSA-F	GCCGAAACAGTCCTGACCTC	This study

4958_EMSA-R	GGTTAGACGGCTCCGCTACTG	This study
4981_EMSA-F	GAGTTGCAGCCTTTCCTTGCC	This study
4981_EMSA-R	CGCGGCGAACAACTTCCTTAC	This study
1081_EMSA-F	CATCATCAACCTGCGCTGATGAC	This study
1081_EMSA-R	GTCCATCGAATCCTCCAGGCA	This study
1440/41_EMSA-F	GGCGAAGGACATCAGGGTACT	This study
1440/41_EMSA-R	CGGCCATGGGAATTCTCCAT	This study
pelA_EMSA-F	GTTTCCGGTGGCAACGTCG	This study
pelA_EMSA-R	GTTGACCTGCAAAGCGTCACG	This study

LasR

**Primers**	**Sequence (5'-3')**	
0861_EMSA-F	GGCTCTACGGACATCTGCAACAG	This study
0861_EMSA-R	CTGACTGCATCACCTGTCGGC	This study
1131/32_EMSA-F	GTCGTGGACACGCCAGGATC	This study
1131/32_EMSA-R	CGTTAGGAAACAACGTTGAGAGGC	This study
1250_EMSA-F	GTCTGACGCGCTGACACGG	This study
1250_EMSA-R	CATCGGATAACACGGCGAACG	This study
2345/46_EMSA-F	CTTCGACGATCCGAGTGGGTG	This study
2345/46_EMSA-R	GTCTGTTTGCACAAGCCGTGC	This study
3141_EMSA-F	CATTCGCTTATAGCGGCGCG	This study
3141_EMSA-R	CATGATGGCACGTTGGTGTCG	This study
3347_EMSA-F	CAGTGATGGCCATGGAAGTCTCC	This study
3347_EMSA-R	GACTTTCGCCCACTGCCCAG	This study
3689/90_EMSA-F	GTAGCGGATGGTCTCCACCG	This study
3689/90_EMSA-R	CAGCAAGTGTCGGCGTGTTC	This study
4067_EMSA-F	GTGGCTGAACGACATGCGAC	This study
4067_EMSA-R	CCAGCAGGGAAGCGGTAAGC	This study
4175_EMSA-F	GCAACTTCCTCAATCGTTCGCG	This study
4175_EMSA-R	GAAGCAAACCGAAGGCTCTGC	This study
4787/88_EMSA-F	CGTCGGTCAGATCACGCATG	This study
4787/88_EMSA-R	GCTGTCAGCTTAGTCGAGCACC	This study
5454/55_EMSA-F	GGAAGCGGGCCTTTTATCTGATG	This study
5454/55_EMSA-R	CGCGACCTGTTCGATCAATAGAACAG	This study
as2779_EMSA-F	GCTGTTCTGGATACCGCAACG	This study
as2779_EMSA-R	CTTCGACCCGAAGCGCTGAG	This study
PhcnA_EMSA-F	ACCGTCGCTGTCGGTGAAC	This study
PhcnA_EMSA-R	TGCCCTTTCATCCGTGAGAGAGA	This study
rsaL_CDS-F	GCTGGCGATCGGTAATTTGCC	This study
rsaL_CDS-R	CATGGCCTTCCGGGCAAAG	This study

Primers-real time rt-PCR primers

ErdR

**Primers**	**Sequence (5'-3')**	
0045_RT-F	CAAGAAAGCACTACTGCCGC	This study
0045_RT-R	ACGCCATCGGAAAAGATCCC	This study
50S_RT-F	ATCGGGCTGTAGGTCTGGAA	This study
50S_RT-R	GAACAAAGAGATCCCGGCGT	This study
0508_RT-F	ACAAGATCAGCGGGACCAAG	This study
0508_RT-R	AACCCAGGCTGTTCTTCTCG	This study
1182_RT-F	CCAGATCACCAGCAGGCTTT	This study
1182_RT-R	CTGATTCATTGCCGGACCCT	This study
1183_RT-F	GCCAACTGATGCTGTGCTTC	This study
1183_RT-R	GCCGAGCTTCTCCATCTTGT	This study
1755_RT-F	ATGTCGAGATTGTCGCGTCC	This study
1755_RT-R	CGCCCAGTATGGCGTAGAG	This study
2623_RT-F	AATCCTTGAAGGCACCCTCG	This study
2623_RT-R	ATGGGCGTCAAGAAGATCCG	This study
2624_RT-F	ACCAGCGTATCGAAGACGAC	This study
2624_RT-R	TGCGCGAACCTCTTTCTCTT	This study
2679_RT-F	CGAAGGGGAACGGCAGATT	This study
2679_RT-R	CTTCTACGAGGAAGTCCGGC	This study
2680_RT-F	TGGTGTCGAAGGCGGAAAG	This study
2680_RT-R	GACGCAAGCTGTTCAGGGAT	This study
3235_RT-F	TGCGCCTCCTTGAGGATTTC	This study
3235_RT-R	TCCTGCTGATCGCTTTCCAG	This study
3266_RT-F	ACGGCACCGTAAAATGGTTC	This study
3266_RT-R	GACAACTTGTACTTCGTCGGC	This study
3437_RT-F	ACAACCTGACCCTGTCGTTC	This study
3437_RT-R	GGTTGTCCAACAGGTAGCGA	This study
3568_RT-F	CACCCTGTGCTCTTCGATCA	This study
3568_RT-R	GTCCGCACTATCTACGGCAT	This study
3766_RT-F	GGCGAACTCGGTATTGTCGT	This study
3766_RT-R	TCAGCGCCTTCTTCGGTATC	This study
3986_RT-F	CGCTGTTTCAACGAGACGTG	This study
3986_RT-R	GTTTGAGCGAAGCGGAGATG	This study
3987_RT-F	GATCATCCACTGCGAGGCTT	This study
3987_RT-R	GACGAAGGTGTCCATGGTGT	This study

FleQ

**Primers**	**Sequence (5'-3')**	
0359_RT-F	GAACCTGTTTCGCACCACC	This study
0359_RT-R	GGCGATCAGGAAGGGACG	This study
0362_RT-F	TCAACTGGTCCTTGCTCTCG	This study

0362_RT-R	GACGTCTGCGAACCCGAAT	This study
2392_RT-F	CCAGCCGCTCAGTTGATCTT	This study
2392_RT-R	TGGAGGGCATCCAATGGTTC	This study
2393_RT-F	CAACGCTTCGGTGAATACGC	This study
2393_RT-R	CAGTGACGTTGCGGATTTCG	This study
2619_RT-F	GGAAGGTACTGTCGTCGACAC	This study
2619_RT-R	CCTTGCTCAGGTCGTAGGG	This study
2653_RT-F	TCATGCCTTCGGTGAACAGG	This study
2653_RT-R	TCGATGTCAGCTACAACCCG	This study
2654_RT-F	GCTGGAATCCGAGTCGATGA	This study
2654_RT-R	ACCTTGCCATCCAGTTCGAG	This study
2955_RT-F	GCCTGTTCCTCGACTGTTCA	This study
2955_RT-R	CGCAGTGTCATCCAGGTCAT	This study
4583_RT-F	TACCTCACGGCAAGACCTTC	This study
4583_RT-R	GCAGACCTCGATGAAGTGGT	This study
4958_RT-F	GAACGTACCTTCCAGCGCA	This study
4958_RT-R	TCGGTGACCTTCTGTGCATC	This study
4981_RT-F	CATGATCTCGCAACCCTGGA	This study
4981_RT-R	GCGATCCTGGTGTTCATCGT	This study
1081_RT-F	TGGTGGACCGGATCAAACTG	This study
1081_RT-R	ATCGCGGTCATCTCTTCCAC	This study
1440_RT-F	CTGCTCGAACCAGACTTCCT	This study
1440_RT-R	ATCGAGACTTCCCTCGAGCA	This study
1441_RT-F	CCTGAGCCCAGTAACGACAA	This study
1441_RT-R	CAAAGGCTTGCCGTCTTCG	This study

LasR

**Primers**	**Sequence (5'-3')**	
0861_RT-F	ACGATCTGCTTCGAGGTCAC	This study
0861_RT-R	AGCTGCCATCGATCTTCAGG	This study
1131_RT-F	CAGACCAGCCAGAATGACGA	This study
1131_RT-R	GAGAAGTTTCACTGGAGCAGC	This study
1132_RT-F	CCGCGCTACTACGAGCTTTA	This study
1132_RT-R	GGCGTAGAACGACAGTTGGA	This study
1250_RT-F	GCTCTCCAGGATGGTTTGCT	This study
1250_RT-R	CCCAGGTCGTAGCCACTG	This study
2345_RT-F	CGACCTTCACCAGGTTGGAA	This study
2345_RT-R	AAGCCATGTACCTGTCCTGC	This study
2346_RT-F	GAAACCGCACCCAAGGATCT	This study
2346_RT-R	GCGGGTGAAGAGTTCCAGTT	This study
3141_RT-F	GAAGCGGATGAACAGGGGAA	This study
3141_RT-R	TAAAGCTCCTGGGATTGCCG	This study

3347_RT-F	GAAGTTGGAGATGGCGAGGA	This study
3347_RT-R	GGATTTCCGCGACGCCTA	This study
3689_RT-F	ACGGATTTCCTCCTGGGTCA	This study
3689_RT-R	GGTGAGCTGGCGAAGAGAA	This study
3690_RT-F	CAAGTTGCCGCAATTCGTCC	This study
3690_RT-R	AGACCGTTGAACACCACCAG	This study
4067_RT-F	CCGCTGCGGATATTCAAGGA	This study
4067_RT-R	GTCGGCGAACATGTAGGTGA	This study
4175_RT-F	CGACCTGACAACCCTCGAAT	This study
4175_RT-R	GCTGGCCTCGAAGATTTCCT	This study
4787_RT-F	CGTACGTAGCGCAGGGAAT	This study
4787_RT-R	TTTCCCCCATTCCGCACAG	This study
4788_RT-F	CGAAATTCCGCCGAAAGCC	This study
4788_RT-R	TGCGAGCCTTGTTCCACAG	This study
5454_RT-F	CTGCCCGGAACAGACGTTAT	This study
5454_RT-R	GCAGAAGGACAGCTTCGTGA	This study
5455_RT-F	AATCCTCGAATCGCTGGGAC	This study
5455_RT-R	GTCGGGAATCGTCTCGAACT	This study
as2779_RT-F	GAACCCCAAGGGTATTTCCCG	This study
as2779_RT-R	CGGGGCTATTCGCCCTTC	This study

PCR primers for first-generation sequencing of promoter mutations

**Primers**	**Sequence (5'-3')**	
PlasB-F	CGTTGTGGAATTGCTCGTAGCG	This study
PlasB-R	GCAACTGATGATCGTCCACATGGC	This study
PmvfR-F	GCGTCATAGTCGCTACACCTGAAG	This study
PmvfR-R	GGAGGAAATCGAACCGGAGGC	This study
Ppa3904-F	CGTCAACCTCAGCCTGATGGAAG	This study
Ppa3904-R	CAGTACGTTGGCGATGACGTTG	This study
PpvdS-F	CAGAACAATTGCAGGGAAACGGC	This study
PpvdS-R	CGGGTAGACAGTTGTTCCGACATG	This study
PambB-F	GTAGCGTCCCAGCAGTACCAG	This study
PambB-R	GATCTCCACGGTATTTGCCCTGATC	This study
PrsaL-F	CTATAGAGTGGGCTGACTGGAC	This study
PrsaL-R	CTTCACTTCCTCCAAATAGGAAGCTGAAG	This study
PrhlI-F	GACCAAGTCCCCGTGTCGTG	This study
PrhlI-R	CGACCAGCAGAACATCTCCAGCTTC	This study
Ppa4677-F	GGAATGTCGAGATAGGTGGTGCG	This study
Ppa4677-R	CGCCTGCTCGAACACCTGATG	This study

Table S2. Linkers used in the study

name	Linker type	DNA sequence	Amino acid sequence
Gly2	Flexible	GGTGGT	GG
Gly4	Flexible	GGTGGTGGCGGA	GGGG
Gly8	Flexible	GGTGGTGGCGGAGGCGGCGGTGGA	GGGGGGGG
(G4S)1	Flexible	GGTGGTGGCGGATCG	GGGGS
(G4S)3	Flexible	GGTGGTGGCGGATCGGGAGGCGGTGGCTCCGGCGGCGGTGGATCG	GGGGSGGGGSGGGGS
(G4S)5	Flexible	GGTGGTGGCGGATCGGGAGGCGGTGGCTCCGGCGGTGGAGGCTCCGGCGGCGGTGGATCGGGAGGCGGTGGCTCC	GGGGSGGGGSGGGGSGGGGSGGGGS
(EA3K)1	Rigid	GAGGCCGCTGCGAAG	EAAAK
(EA3K)3	Rigid	GAGGCCGCTGCGAAGGAAGCAGCTGCCAAAGAGGCTGCTGCTAAG	EAAAKEAAAKEAAAK
(EA3K)5	Rigid	GAGGCCGCTGCGAAGGAAGCAGCTGCCAAAGAAGCAGCTGCCAAAGAGGCTGCTGCTAAGGAAGCGGCAGCCAAG	EAAAKEAAAKEAAAKEAAAKEAAAK

References

1. Hmelo, L.R. *et al.* Precision-engineering the *Pseudomonas aeruginosa* genome with two-step allelic exchange. *Nature protocols* **10**, 1820-1841 (2015).
2. Han, J. *et al.* Simultaneous Visualization of Multiple Gene Expression in Single Cells Using an Engineered Multicolor Reporter Toolbox and Approach of Spectral Crosstalk Correction. *ACS Synthetic Biology* **8**, 2536-2546 (2019).
